# A robotic shower system: Acceptance and ethical issues

**DOI:** 10.1007/s00391-017-1345-9

**Published:** 2017-12-11

**Authors:** Barbara Klein, Inga Schlömer

**Affiliations:** Frankfurt University of Applied Sciences, Nibelungenplatz 1, 60318 Frankfurt am Main, Germany

**Keywords:** Independent living, Hygiene, Activities of daily living, Nursing homes, Elderly, Selbstständiges Leben, Hygiene, Aktivitäten des täglichen Lebens, Pflegeeinrichtungen, Ältere

## Abstract

**Background:**

Being able to maintain personal hygiene plays a crucial role for independent living in old age or when suffering from disabilities. Within the European project ICT Supported Bath Robots (I‑SUPPORT) an intelligent robotic shower system is being developed, which enables patients to shower independently at home or in institutionalized settings.

**Objective:**

The aim of this contribution is the identification of ethical issues in the development of a robotic shower system utilizing the model for the ethical evaluation of socio-technical arrangements (MEESTAR).

**Material and methods:**

In I‑SUPPORT a variety of concepts and methods are implemented in order to achieve technology acceptance such as user-centered requirements analysis, usability-tests and analysis of sociocultural and ethical aspects. This article reports the analysis of focus groups with 14 older adults and 9 professional caregivers utilizing MEESTAR as a heuristic approach for analyzing sociotechnical arrangements and identifying ethical problems.

**Results and discussion:**

The MEESTAR procedure was adapted to the research question and client groups and implemented as a discursive method. This gave an insight into the meaning and background of ethical aspects and also a deeper insight into nursing processes as well as the requirements which the system should fulfil. Shortcomings are that the ethical dimensions are not everyday language and the time restrictions. In the next step a standardized assessment instrument will be developed and piloted.

## Zusammenfassung

**Hintergrund:**

Die Fähigkeit, die persönliche Hygiene beibehalten zu können, ist ein entscheidender Faktor für ein selbstständiges Leben im Alter und bei Behinderung. Im Projekt ICT Supported Bath Robots (I‑SUPPORT) der Europäischen Union wird hierfür eine intelligente robotische Duschhilfe entwickelt, die es ermöglicht, sicher und selbstständig zu Hause und in Pflegeeinrichtungen zu duschen.

**Ziel der Arbeit:**

Ziel des Beitrags ist die Identifikation ethischer Probleme im Rahmen der Entwicklung einer robotischen Duschhilfe anhand des Modells zur ethischen Evaluation sozio-technischer Arrangements (MEESTAR).

**Material und Methoden:**

In I‑SUPPORT werden unterschiedliche Konzepte und Methoden mit dem Ziel der Technologieakzeptanz eingesetzt, wie z. B. nutzerzentrierte Anforderungsanalysen, „usability tests“ sowie die Analyse sozio-kultureller und ethischer Aspekte. Der vorliegende Beitrag beschreibt die Auswertung von Fokusgruppen mit 14 älteren Menschen und 9 Pflegefachkräften, bei denen MEESTAR als heuristisches Verfahren genutzt wurde, um die sozio-technischen Szenarien zu analysieren und ethische Probleme zu identifizieren.

**Ergebnisse und Diskussion:**

Das MEESTAR wurde methodisch an die Untersuchungsfragestellung und -gruppen angepasst und als diskursive Methode eingesetzt. Dieses ermöglichte einen Einblick in die Bedeutung und Hintergründe der ethischen Aspekte sowie einen vertieften Einblick in das pflegerische Handeln und die Anforderungen, die das System erfüllen soll. Als „Schwäche“ erwiesen sich die nicht alltagssprachlichen Begriffe der ethischen Dimensionen und die zeitlichen Anforderungen. Im nächsten Schritt wird ein standardisiertes Assessmentinstrument entwickelt und pilotiert.

## Background and aims

**Fig. 1:**
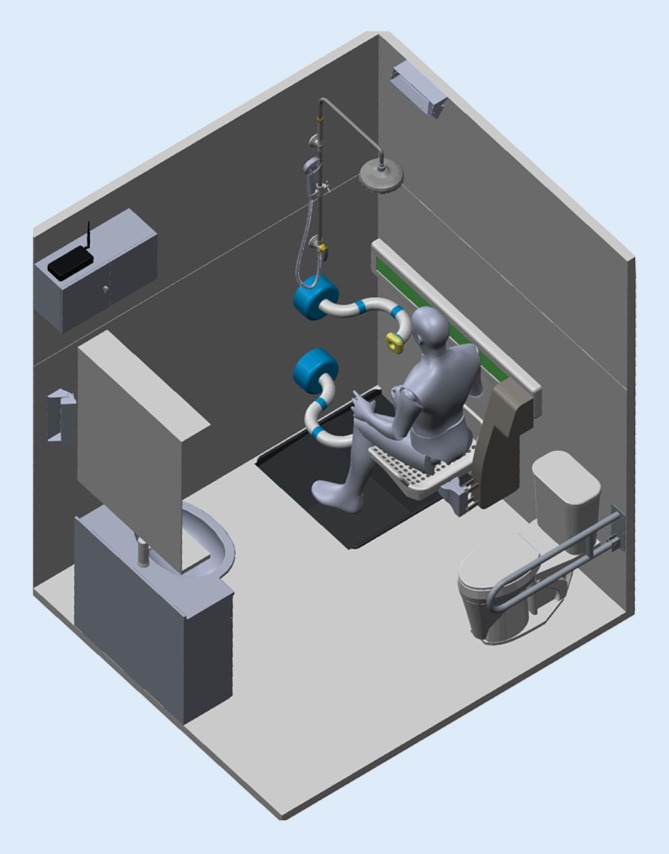
Rendering of the I‑SUPPORT robotic shower system. (image source: The BioRobotics Institute, Scuola Superiore Sant’Anna, IT, with kind permission)

A crucial factor for independent living is the ability to maintain personal hygiene. In Western culture personal hygiene is one of the most sensitive and intimate subjects and is usually carried out in privacy. Relying on support from relatives or home care services is often accompanied by feelings of shame and helplessness [1, 9]. In this situation an automated showering support system can foster independent living with dignity and may relieve stress of family members or professional caregivers. The European research project I-SUPPORT is developing an intelligent robotic shower system which enables elderly or disabled individuals to shower independently at home and in care homes. This robotic shower system (Fig. 1) consists of a motorized chair to enable safe stand-to-sit and sit-to-stand transfer, a robotic shower arm utilizing soft robotics, and various human-robot interaction modalities. The system can be operated by speech input, by gestures and also by remote control. Pressure sensors are implemented to detect contact with the body of the user.

The research on service robots in care settings has predominantly focused on the development and effectiveness of robot implementation and ways to achieve acceptance. In this context different technology acceptance models [3, 4] and the Medical Research Council (MRC) framework propose [2, 7] approaches dealing with this complex subject. Only recently are ethical considerations becoming increasingly more relevant [22, 24]. Various approaches have been proposed for considering ethics in the development of age appropriate assistive technologies. Sorell and Draper proposed an ethical framework for the design of “carebots” [20] and addressed six ethical values that should be considered in the design for older adults. Van Wynsberghe presented the care-centered framework and the care centered value sensitive design (CCVSD) methodology [21]. Kricheldorff and Tonello proposed the interdisciplinary dialogue instrument for technology use in old age (IDA) [13]. This tool provides a guideline for enabling and arranging a dialogue on ethical considerations focusing on eight basic values on a first level. On a second level these values are specified by considering specified environmental conditions and adding corresponding aspects. General approaches, such as Value Sensitive Design (VSD) or methods that focus on contiguous topics, such as the Manual MethoTelemed (MAST) that focus on telemedicine, address the ethical considerations [10, 11]. Manzeschke et al. defined the model for the ethical evaluation of socio-technical arrangements (MEESTAR) that serves as an ethical evaluation tool [15]. As a heuristic instrument it provides guidance on ethical considerations and the decision to use assisting systems. For this study it was identified as a particularly suitable guide for a structured dialogue concerning a socio-technical scenario in order to analyze its use, identify moral problems, and find adequate solutions. The application of the MEESTAR will be addressed in detail in the Methods section.

## Material and methods

**Fig. 2:**
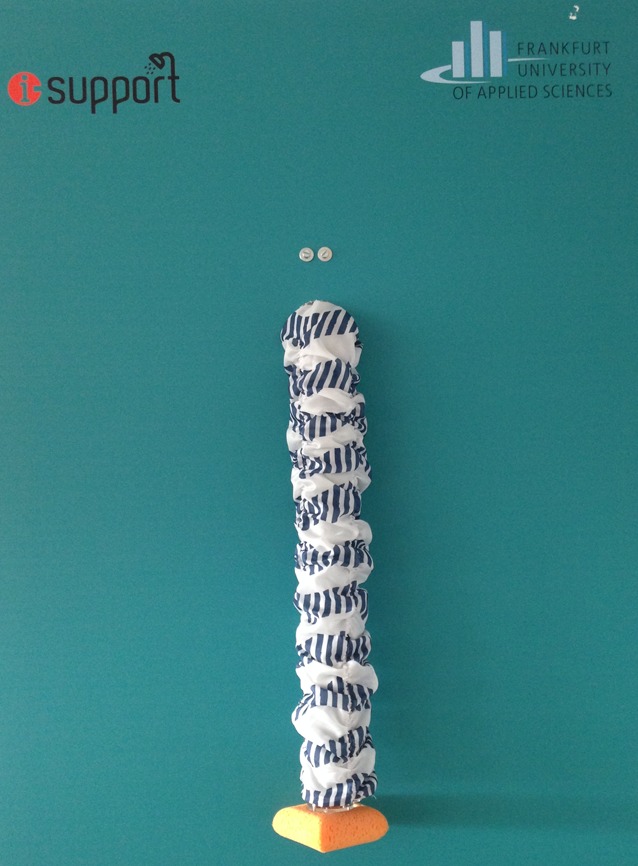
Mock-up of the robotic shower arm mounted on cardboard [6]

User involvement in technological development plays a crucial role in technology acceptance. In I‑SUPPORT primary and secondary users are involved in all relevant stages of technological development. Primary users (PU) identified for the robotic shower system are people not able to wash themselves, mainly older, frail persons. Secondary users (SU) are professional caregivers who carry out these tasks either in a private or institutionalized setting. Both user groups were involved from the start of the project contributing to requirement analysis [12] and giving an early feedback on first renderings (Fig. 1) and mock-ups (non-functional models; Fig. 2).

### The MEESTAR model

Ethical questions play a crucial role in technological and also in robotic development and is even more important in the healthcare sector dealing with vulnerable groups. In order to identify the most important ethical issues the MEESTAR was utilized as an analytical instrument for discourse.

**Fig. 3:**
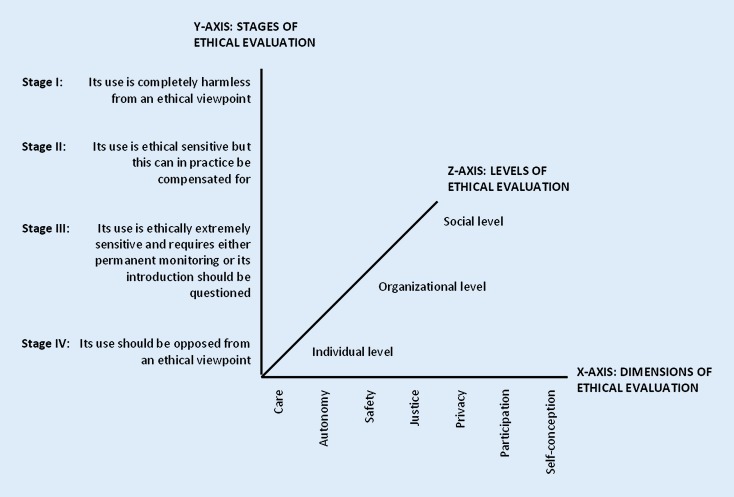
MEESTAR: a model for the ethical evaluation of socio-technical arrangements. (After [15])

Fig. 3 shows the MEESTAR with its different dimensions.

The MEESTAR is based on three axes. Manzeschke et al. suggested seven ethical dimensions which are presented on the X‑axis (care, autonomy, safety, justice, privacy, participation and self-conception; [15]). The Y‑axis presents the four assessment-stages how the ethical dimensions can be perceived (harmless until the use should be opposed from an ethical viewpoint). The Z‑axis represents three different perspectives (individual, organizational, social) to discuss the ethical dimensions. For each ethical dimension two up to four key questions are provided [15]. For an ethical evaluation MEESTAR supports discussions with content and topics. Although MEESTAR was developed for age-appropriate technologies (i. e. mainly sensor-based monitoring systems) it was assumed that these and other ethical dimensions could play a crucial role for the robotic shower system; however, an assumption was that the ethical dimensions of MEESTAR were not equally important to the system and that there might be other ethical issues crucial to the system. The approach was adapted in order to identify further essential ethical issues.

### Study design

Different methods are applied throughout the I‑SUPPORT project. In the beginning of the project interviews with 30 PU and 15 SU took place analyzing the requirements for the shower system and discussing renderings to gain first impressions on ethical questions. Based on these results, four focus groups were organized to discuss ethical issues with respect to the I‑SUPPORT system utilizing MEESTAR. The purpose of the first three focus groups was to assess important crucial ethical dimensions with respect to a robotic shower system as well as to gain feedback on the advanced rendering and mock-ups for a more detailed feedback to technical partners. Of the focus groups two were active in the Independent Living Centre of Frankfurt UAS^1^: one with PU living independently in their private home and one with SU. The work of the third focus group with PU and SU took place in a nursing care home where the PU were residents and SU were nursing care staff of the care home. The reasons for this location were that the robotic system is also meant to be for institutional settings, such as nursing care homes; PU in care homes are usually more mobility restricted and have experienced more functional loss compared to PU able to come to the Independent Living Centre. The assumption was to obtain a deeper insight into the requirements of this environment and the impact on the I‑SUPPORT system. This was also a reason for the composition of this focus group with PU and SU as they could contribute from their different perspectives of daily practice. After the experiences in the three focus groups the research team decided to have a workshop on the MEESTAR model carried out by one of the developers. As a consequence, the team decided to undertake an additional fourth focus group with PU in a nursing care home. The aim was to utilize an adapted MEESTAR focussing particularly on the individual perspective of PU as this seemed to be most relevant at that stage of the technological development.

All participants in the focus groups gave informed consent. The focus groups were voice recorded. In order to analyze the collected data, audio recordings were transcribed. The qualitative analysis followed Mayring’s process of summarizing, explication and structuring [17]. Data derived from the transcript of each focus group was coded based on thematic categories (e.g. ethical dimensions) utilizing the MAXQDA software (VERBI Software, Consult – Sozialforschung GmbH, Berlin, Germany) [14].

Table 1 gives an overview of the composition of the 23 participants in the focus groups.

**Table 1:** Overview of the participants of the four focus groups

	Primary users (PU)		Age range (years)	Secondary users (SU)		Age range (years)
	Male	Female		Male	Female	
Focus group PU	2	5	62–84	–	–	–
Focus group SU	–	–	–	3	3	36–63
Focus group MG	3	1	82–90	1	2	37–44
Fourth focus group	3	1	56–82	–	–	–

*PU* primary users, *SU* secondary users, *MG* mixed group

The schedule of the focus groups started with an introductory round, a presentation on the project and an introduction of the mock-up and the rendering (Figs. 1 and 2, [5, 12]). In the first three focus groups this was followed by a discussion on benefits, desirable functions, operability and usability requirements. The importance of the different ethical dimensions of the MEESTAR system was then discussed, additional concerns and values recorded and participants assessed from their perspective the three most important dimensions of MEESTAR with adhesive dots, which were then added together to give a total assessment. The goal of the fourth focus group with PU was to discuss the meaning of the ethical dimensions for the robotic shower system and assess the potential stage of danger/harmlessness from their point of view.

## Results

The PU rated the robotic shower system for all ethical dimensions with stage II: “Its use is ethically sensitive but this can be in practice compensated for”. The discussion in all focus groups revealed a range of suggestions for a safe environment and desirable features for the system. The analysis of the three focus groups gave an insight in the different perceptions of PU and SU on the importance of the different ethical values. Participants distributed three sticky dots on those ethical dimensions they assumed to be the most important for the system. These dots were added together for PU and SU. Table 2 gives an overview of their distribution.

**Table 2:** Results of assessing the importance of ethical dimensions for the development of I‑SUPPORT based on the allocated number of sticky dots

	*N*	Autonomy	Safety	Privacy	Self-conception	Justice	Care	Participation
Focus group PU	7	7	1	3	6	0	2	2
Focus group SU	6	6	5	2	0	5	0	0
Focus group MG: PU	4	2	2	1	1	1	2	0
Focus group MG: SU	3	2	3	2	0	1	1	0
Total PU	11	9	3	4	7	1	4	2
Total SU	9	8	8	4	0	6	1	0
Total	20	17	11	8	7	7	5	2

*PU* primary users, *SU* secondary users, *MG* mixed group

The PU scored highest on autonomy and self-conception and SU scored high on autonomy, safety and justice. Participants addressed ethical issues throughout the workshop. The most important findings are presented in the following.

### Ethical dimensions: autonomy, privacy and choice

One of the surprising results was that PU, i.e. people with functional loss, have a positive attitude towards a robotic shower system if it enables them to take care of their personal hygiene. It was considered to be better to use technology instead of a human caregiver because of the preferred privacy of this intimate process. Autonomy was equated with a maximum of decision-making and freedom of action. The robotic shower system should provide a variety of choices, different settings and individualized programs according to user preferences, e. g. both user groups emphasized the possibility of taking a shower when and for how long they wished.

OK, I say people who are still very independent, they can of course also choose the time when to shower. So there isn’t a fixed time set: now I have to take a shower. I can also have a shower in 2 hours time (Transcription mixed group PU: 223–223).

These arguments have an underlying additional ethical theme: choice. The possibility to choose is strongly related to autonomy [8]. Often choosing out of a range of options is one of the last parts of autonomy when experiencing functional loss. This also includes the choice to decide whether you want to use a robotic shower system as well as design and functional aspects e. g. the choice of colors or different auxilliaries, such as sponges and washcloths.

Although professional caregivers rated autonomy high, they saw themselves in a multifold dilemma: taking care of the personal hygiene process does not only mean that caregivers bring a person to the bathroom, undress, shower, dry, and dress the care recipient. This process has veiled aspects which seem to be essential in professional care. These aspects are A) communication, B) promotion of still existing resources of the care recipient and C) monitoring health changes.

A. The importance of communication is stressed by the fact that the professional caregiver is often the only person during the day the care recipient meets and can talk to. Communication can also be a (kind of) reciprocal process where caregiver and recipients mutually act and react, thus establishing a relationship which can contribute to their mutual well-being; however, mutuality has its limitations as the relationship is characterized by disparity as the care recipient depends on the caregiver.
B. One of the main aims of communication in the care process is to promote still existing resources of the care recipient. Motivation to perform even small tasks, such as taking a washcloth and washing the face, can be a process of encouraging communication, maybe accompanied by humor on both sides.
C. Observing the naked body during the shower process allows professional caregivers to monitor the health status. Skin changes can be observed and upcoming pressure sores avoided at an early stage. Thus, the underlying assumption by PU and SU was that professional care has a raison d’être. The encouragement of resources is viewed as an important contribution towards autonomy, a task which might not be fulfilled by a robotic shower system.

### Ethical dimensions: safety and justice

The ethical dimension safety mainly referred to the safe use of the robotic shower system. For SU it was just as equally important as autonomy. The discussion revealed a dichotomy: on the one hand the role as autonomy enhancer for the PU, on the other hand as autonomy restrictor because of the feeling and being responsible for the safety of the PU. The SU also emphasized that to feel safe is highly important and features for the system could be the possibility to trigger alarms or integrating sensors that recognize falls. At no time should the robotic system jeopardize PU; therefore, an emergency stop is highly important. Although, PU did not prioritize safety with their allocation of sticky dots, the discussion in all focus groups revealed safety as being extremely important for them and a sine qua non which concerns not only the system but also the shower environment. The SU rated the ethical dimension justice high and relating mainly to distributive justice. They pointed out that these technologies should be provided to anybody who needs it, and not only for those who can afford it. Other concerns were that the robotic shower system might contribute to savings in staff.

### Ethical dimension: self-conception

The dimension self-conception (highly rated by PU) was discussed with respect to usability requirements (easy to operate, a feeling of happiness when you are able to control the robotic shower system) and non-stigmatizing features of the robotic system (e. g. friendly colors, appealing background noise, and ambience).

### Additional ethical issues: human-robot and human-human relationships

Participants in all focus groups also discussed human-robot and human-human relationships. The opinion of PU and SU differed. Some of the PU would prefer to use the robotic shower system (in order to be independent and have privacy). Other PU prefer to be supported by a human being. A personal relationship and talking during the showering process can play an important role, which was also confirmed by SU who stressed the importance of that aspect especially in home care. Worries with respect to the human-robot relationship were fears of losing the job and concerns on the nature of future work if robots do the work. There seemed to be a unanimous view that relationship work should not be omitted and time saved by technology should be used for essential care tasks which are currently lacking because of scarce staff resources. These results are comparable to the interviews with PU and SU [5].

So I am missing the interpersonal relationship. From my point of view, this is what constitutes care. I completely miss this. As assistive device, sure positive. It is a good idea, but I’m missing the communication or […] on the other hand I know also from my friends, who work in ambulatory/domiciliary services that clients prefer to talk for 5 min instead of getting the back washed. So that is a conflict for me. (Transcription mixed group SU: 406–406).

Another issue of human-robot interaction is that the user is the master of the robotic shower system, i.e. the user can control the robotic system at any time and not vice versa.

Prerequisite will be, of course that I control this thing and not the thing me. Sort of I take the lead and not the machine. This is indeed a very important factor. (Transcription SU: 276–276).

## Discussion

The I‑SUPPORT project is dealing with a robotic system alleviating an intimate basic activity of daily life (ADL). In the search of suitable ethical assessments MEESTAR seemed to be a useful instrument as it includes all stakeholders in the process. The MEESTAR was developed in the context of ambient assistive technologies, so it was assumed that ethical issues could differ with respect to service robots. Hence, the MEESTAR approach was adapted in order to detect the importance of and additional relevant ethical dimensions. The model proved to be flexible enough for the envisaged aims. Weber, one of the developers, confirmed the flexibility of the instrument and stressed the possible differing importance or even conflicting ethical dimensions in MEESTAR^2^, an expansion of MEESTAR [23].

### Strengths

The discourse-oriented approach provided valuable information on core functions of the shower process. The discussions during the focus groups provided not only multiple ideas for the functionalities and desirable features of the system, but also aspects to be considered by the socio-technical arrangements if the robotic shower system is commercially available.

The project iToilet [16, 19] develops an ICT-enhanced toilet system supporting older adults and their care giver in this basic ADL. Comparable to I‑SUPPORT a user-centered approach is applied and PU, SU and other stakeholders are involved in the technological developmental process. Recent publications [18, 19] focussed on user requirements. Ethical issues are considered in the study design of the pilots and as a rationale for the project [18]. Here the ethical dimensions autonomy/independence, empowerment and dignity are mentioned. Similar to I‑SUPPORT, PU and SU are not hesitant to discuss requirements from their perspectives despite the subject being surrounded by even more taboos [18, 19].

### Limitations

In I‑SUPPORT the ethical dimensions were explained in simple language to participants. Facilitators got the impression that these definitions blurred during the course of the focus groups as this is not everyday language. For future work, recourse of everyday language seems to be appropriate, especially if user groups have vision or hearing impairments, are non-academics or not native speakers. The 3‑h time frame was too tiring for some of the participants, even more so for those who lived in a nursing care home. Thus, results reflect the subjective normative assessment of the participants. For the technological development, the insights into the causes and the new ideas created have been valuable.

Future focus groups should take these into account and develop approaches which consider easy to understand language and time constraints as well as often limited (research) resources. For I‑SUPPORT a structured assessment instrument will be developed and piloted in the formative and summative evaluations of the different prototypes by PU, SU and other stakeholders.

A systematic review of argument-based ethics literature on care robots in care of the aged revealed four main ethical approaches [22]: the deontological approach focussing on human reasoning on what is good or should be done. Values described are autonomy and dignity, deception and truth, social isolation and connectedness. The principlist approach as the practical translation of the deontological approach discusses four principles: respect for autonomy, beneficience, non-maleficience and justice. The objective list approach puts forward several “capabilities” or “goods” that can be reached or supported by care practices. The care ethical approaches look at the special care relationship between caregivers and care receivers and widen it to a contextual and political level. In this context MEESTAR could be subsumed to the deontological and principlist approaches, which are viewed as a too restricted approach as “The ethical landscape pertains to human moral agents seeing robot technology merely as a collection of neutral instruments that can or cannot be used to promote the well-being of older adults. Consequently, these approaches seem to lead to an ethical assessment of care robots instead of an open ethical reflection about their use” [22]. Weber defies to assign MEESTAR to a theoretical concept and suggests its use as a discoursive ethical approach [23]. The questions arises whether the way ethical assessment instruments are utilized might contribute to more openness and to possibilities to develop answers on issues, such as what is good care and what impact do “care-robots” have on the concept of care and on society. This question cannot be conclusively answered and here more research is needed also taking into account the different types of robot technology. The approach to include PU and SU from scratch of technological development at least gives the opportunity to get insights into their perceptions and the reasons for that.

## Conclusion

The MEESTAR is a flexible instrument for ethical discourse and also for service robotics. Its application provides a deepened insight into roles and activities around the shower process as well as desirable features to be taken into account in the technological development process. Practical problems, such as easy language and limited time frames for ethical discussions will be addressed by the development of a standardized assessment tool which will be piloted with PU and SU. In order to obtain a deeper insight into the I‑SUPPORT impact on the organizational and social level, focus groups are planned with these stakeholders with a more advanced prototype of the robotic shower system.

## Footnotes

1 The Independent Living Centre at Frankfurt UAS is a permanent exhibition which displays accessible design and a broad range of assistive technologies. The centre is built as a model flat, also displaying an accessible bathroom. Thus, it is well suited for interviews and focus groups for the I‑Support project as the mock up can be displayed in the bathroom to achieve a more “real”-experience (www.frankfurt-university.de/en/independent_living; http://www.youtube.com/barrierefreieswohnen).
